# Molecular mimicry and cancer vaccine development

**DOI:** 10.1186/s12943-023-01776-0

**Published:** 2023-04-26

**Authors:** Maria Tagliamonte, Beatrice Cavalluzzo, Angela Mauriello, Concetta Ragone, Franco M. Buonaguro, Maria Lina Tornesello, Luigi Buonaguro

**Affiliations:** 1 Lab of Innovative Immunological Models, Istituto Nazionale Tumori, IRCCS - “Fond. G. Pascale”, Naples, Italy; 2 Molecular Biology and Viral Oncogenesis Unit, Istituto Nazionale Tumori, IRCCS - “Fond G. Pascale”, Naples, Italy

**Keywords:** Tumor-Associated Antigens, Microbiota, Cancer Vaccines, Molecular Mimicry, T cell cross-reactivity

## Abstract

**Background:**

The development of cancer immunotherapeutic strategies relies on the identification and validation of optimal target tumor antigens, which should be tumor-specific as well as able to elicit a swift and potent anti-tumor immune response. The vast majority of such strategies are based on tumor associated antigens (TAAs) which are shared wild type cellular self-epitopes highly expressed on tumor cells. Indeed, TAAs can be used to develop off-the-shelf cancer vaccines appropriate to all patients affected by the same malignancy. However, given that they may be also presented by HLAs on the surface of non-malignant cells, they may be possibly affected by immunological tolerance or elicit autoimmune responses.

**Main body:**

In order to overcome such limitations, analogue peptides with improved antigenicity and immunogenicity able to elicit a cross-reactive T cell response are needed. To this aim, non-self-antigens derived from microorganisms (MoAs) may be of great benefit.

## Introduction

Tumor antigens can be classified into tumor-specific antigens (TSAs) and tumor-associated antigens (TAAs). TSAs arise from cancer-related nonsynonymous mutations or other genetic alterations, which give rise to mutated peptides presented by HLA only on the cell surface of tumor cells (neoantigens). Consequently, they are non-self-antigens able to elicit a highly specific anti-tumor immune response (reviewed in [1]). However, neoantigens are strictly individual (private) and their identification requires a combination of high throughput omics screening procedures for each cancer patient which currently cannot be applied on large scale [2–4]. Moreover, the efficacy of such a highly personalized approach is possibly reduced by the high mutational rate of tumors, which drives a constant generation of new target mutated neoantigens and a consequent cancer immune evasion.

On the contrary, TAAs are wild-type self-antigens highly expressed on tumor cells compared to corresponding normal cells. Consequently, they can be used for developing off-the-shelf vaccines applicable to all patients affected by same malignancy. However, as they may be expressed also in healthy tissue, they may have major drawbacks [5]. Indeed, TAAs may show low immunogenicity and specific T cells may have low affinity receptors (TCR), which are unable to mediate effective anti-tumor responses [6]. Alternatively, TAA-specific T cells may target the corresponding normal tissues leading to autoimmune diseases [7, 8]. Finally, such T cell populations may be removed from the immune repertoire by central and peripheral tolerance and the efficacy of vaccines based on TAAs may be lost [9]. Therefore, overcoming such limitations is a major goal, given that TAAs represent the most suitable target antigens for developing cancer vaccines on a large scale.

One of the possible strategies is represented by designing analogue peptides in which specific amino acid residues are substituted to improve their antigenicity and immunogenicity (heteroclitic peptides) [10–13]. However, the exploitation of homology between TAAs and antigens derived from microorganisms (MoAs) (molecular mimicry) may provide the most effective results. Indeed, they are abundantly accessible natural non-self-antigens, which do not need any manipulation for improving antigenity or immunogenicity.

## Molecular mimicry

The immune system is able to discriminate between the body’s own “self” cells and foreign “non-self” cells. Therefore, only the latter (i.e. infected cells, extracellular pathogens, cancer cells) are attacked by T cells while normal cells are spared. However, during evolution, microorganisms have been selected for the ability of eluding the host immune’s surveillance.

Indeed, the term “molecular mimicry” was originally introduced in 1964 to define the similarity between antigens expressed by infectious agents and human cells, as cause of the microbial escape from the host immune response and a more aggressive infection [14]. However, already two years earlier, without any experimental evidence of molecular similarity, an opposite implication of the molecular mimicry was postulated. Antibodies cross-reacting to both group A streptococcal cells and human heart tissue were identified in a patient with rheumatic fever suggesting a pathogenetic role in such an autoimmune disease [15]. The phenomena of immune escape and the auto-immunity elicited by the molecular mimicry may appear as contradictory but are the two faces of the same coin, representing the extremes of a biological continuum.

Since then, several reports have shown sequence and structure homologies between pathogen-derived antigens (viruses and bacteria) and cellular self-antigens with the induction of cross-reactive immune responses. This is currently widely accepted as the major pathogenetic mechanism for several autoimmune diseases in humans (reviewed in [16–18]).

**Fig. 1:**
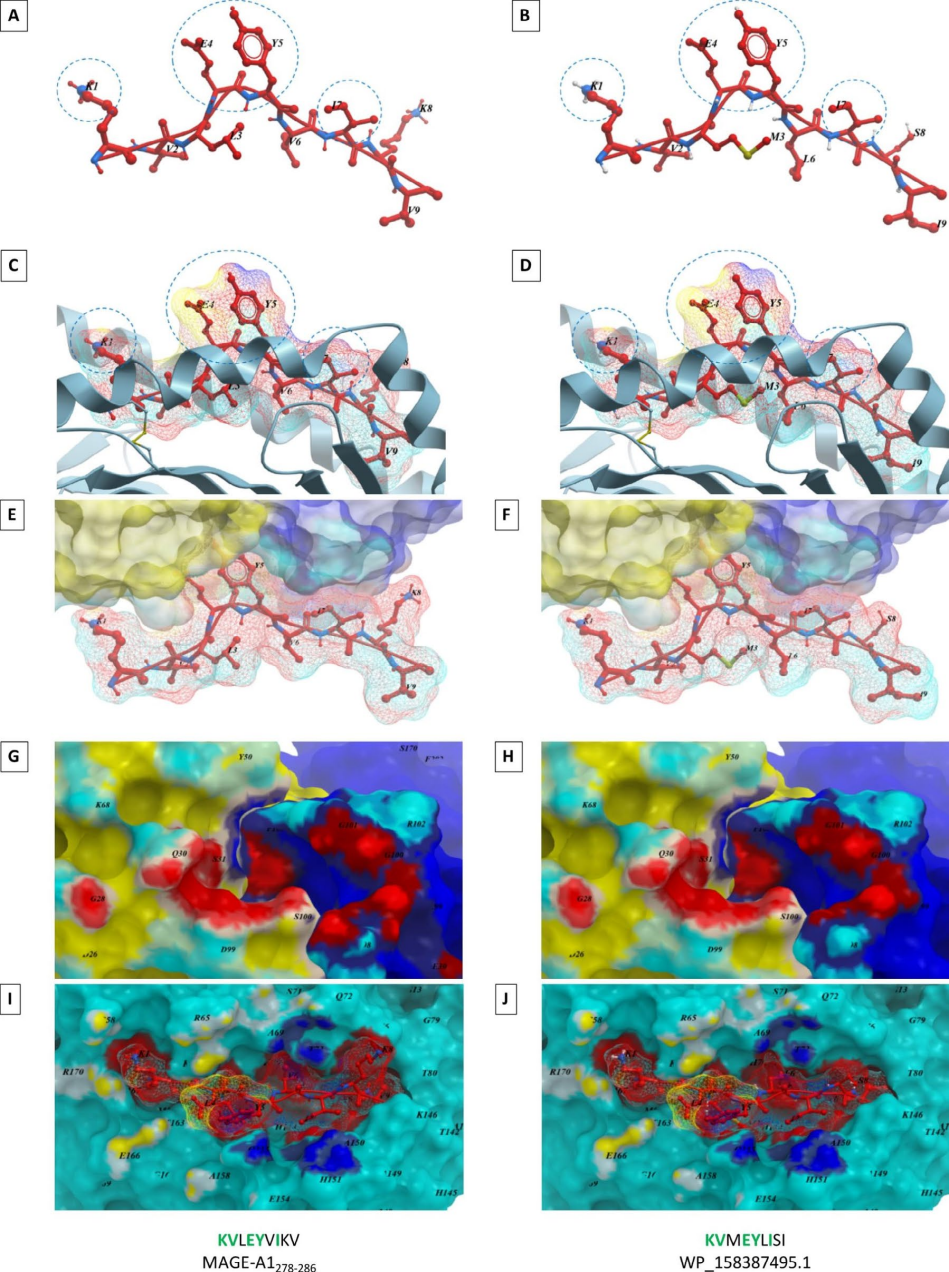
**Molecular mimicry: homology in the TCR facing residues and molecular interaction.** The MAGE-A1_278 − 286_ peptide and the homologous peptide derived from *Faecalibacterium prausnitzii* (WP_158387495.1) are shown bound to the HLA-A*02:01 molecule. The identical residues K_1_, E_4_, Y_5_, I_7_ are presented to the TCR αβ chains with the same conformation (**A** – **D**). The contact areas in red between the two peptides and the TCR αβ chains are identical (**E** - **H**). The areas of contact between the HLA molecule and the TCR αβ chains are identical (yellow and dark blue areas) (**I**, **J**)

Interaction between the peptide and the MHC class-I molecule has been clarified by crystallographic analysis. The peptide is anchored to the MHC-I groove through the “anchor positions” (p2 and p9), with highly conserved amino acid residues specific to each HLA allele [19–22]. The two-to-four central positions are facing upward towards the TCR and are characterized by epitope-specific “TCR facing” residues. Consequently, the molecular mimicry is strictly dependent on the latter residues which should be either identical or with the same chemical properties (Fig. 1, A - H). In particular, three categories of molecular mimicry have been defined according to the similarity between microbial and host antigens. Type 1: complete identity of amino acid sequences in different protein molecules; type 2: structural similarities independent from the amino acid sequence identities; type 3: dissimilar chemical structures recognized by the same antibody [23].

## Cross-reactive immunity

Apart from the homology between TCR facing residues of two epitopes, the molecular mimicry would not play any role in human diseases if T-cell receptors (TCRs) would not be able to cross-react with different peptides when complexed to MHC (pMHC complexes).

For several years, the *clonal selection theory* was widely popular, proposing that each T cell is specific for a single antigen (the so-called “one-clonotype-one-specificity” paradigm) [24, 25]. However, starting from the late 90’s, the theory was questioned based on a simple numerical evidence. Indeed, in a human body are present a maximum of 10^12^ circulating T cells which should recognize > 10^15^ potential foreign peptides; moreover, of these T cells less than 10^8^ have a naïve phenotype expressing unique TCRs which cannot undergo affinity maturation [26]. The implication is that the entire pool of naïve T cells must express from the beginning, without possibility of subsequent adaptation at protein level, TCRs able to recognize the entire spectrum of > 10^15^ potential foreign peptides.

Consequently, if the clonal selection theory would be valid, a huge number of pMHC complexes (approximately 1 × 10^7^) would not be “seen” by T cells. Therefore, if each TCR would not be able of responding to a great number of different pMHC complexes, thousands of antigens (and therefore pathogens) would have taken advantage of such a “blind spot” with fatal consequences for the host during the evolution [27–30].

The TCR-pMHC interaction is based on a general rule in which the CDR1α, CDR1β, CDR2α and CDR2β elements of the TCR (non-rearranged, germline-encoded) get in contact with the MHC molecule, whereas the somatically rearranged CDR3α and CDR3β loops get in contact with the peptide [31–33]. In such interaction scheme, all known TCR–pMHC-I structures are characterized by the contact between the TCR and the three highly conserved residues in the MHC class I at positions 65, 69, and 155 (the “restriction triad”), representing the minimal docking framework for the MHC-I restriction [34] (Fig. 1, I and J).

However, not all TCR-pMHC complexes fall in such a rule [35] and pairwise interactions between a TCR and a peptide–MHC complex widely varies between individual TCRs [36]. This leads to an extensive T cell cross-reactivity as shown by evidences that a broad T cell repertoire can be selected by a single peptide [37] and, conversely, the resulting T cells can be activated by unrelated peptides [38].

## TAAs and viral antigens

The first evidence for homology between a TAA (namely MART-1_27−35_ AAGIGILTV) and viral antigens was reported by Loftus et al. [39]. In particular, peptides from the Herpes simplex virus-1 (HSV-1) (GIGIGVLAA), the Herpes simplex virus-2 (HSV-2) (GAGIGVAVL), the Pseudorabies virus (IAGIGILAI) and the Adenovirus 3,7 (LIVIGILIL) were identified with PIR Protein database. The peptides show high homology or amino acid conservation at positions P_3_ – P_7_ and they all sensitized TAP-deficient T2 cells for lysis by anti-MART-1 effector T cells. Moreover, cross-reactivity of TCRs was confirmed by cold target inhibition experiments showing that T2 cells pulsed with the HSV-1 peptide inhibited lysis of ^51^Cr-labeled T2 cells pulsed with the MART-1_27−35_ peptide. Independent studies showed that healthy subjects have Melan-A-specific cells in peripheral blood mononuclear cells (PBMCs) with a naïve CD45RA^hi^/RO^−^ phenotype, whose specificity was confirmed by their ability to kill T2 target cells pulsed with the Melan-A_26–35_ peptide [40]. The meaning of such finding was deeply investigated on 37 A2/Melan-A multimer^+^ CD8^+^ T cell clones derived from a healthy donor tested against 71 Melan-A-related sequences. 10 of such sequences were derived from human viruses of viral origin and 7 of them were cross-recognized by A2/Melan-A multimer^+^ CD8^+^ T cell clones leading to cell lysis of Melan-A-expressing melanoma tumor cells [41]. Such findings were further confirmed on 7 Melan-A-monospecific polyclonal cytotoxic T lymphocytes (CTL) cell lines as well as Melan-A-specific CTL clones from melanoma patients [42].

More recently, tumor-infiltrating T cells in human lung cancer have been shown to cross-react with a tumor antigen derived from the TMEM161A protein and an antigen derived from the Epstein-Barr virus (EBV) latent membrane protein 2a. The EBV-specific T cells show an effector phenotype suggesting that the cross-reactivity may play a role in controlling cancer progression, overcoming tolerance for TAA [43].

We have previously shown in a cohort of HCC patients that predicted mutated neoantigens with high affinity to HLA may show > 50% sequence similarity to pathogens’ derived epitopes with identical central residues exposed to bind the TCR. In particular, for each paired epitopes, the bioinformatics modelling shows very similar epitope conformation when bound to the HLA molecule with an almost identical pattern of contact with the HLA residues. Moreover, a comparable spatial conformation of the residues protruding towards the TCR is observed. PBMCs from a long-term surviving HCC patient cross-reacted against both the tumor-specific mutated neoantigens and the paired pathogen epitopes. The most potent reaction was observed against the mutated neoantigen with high homology to the vaccinia virus derived epitope, suggesting that the memory T cell elicited by the vaccinia virus vaccine may have played a role in the observed response [44]. More recently, we have performed a complete sequence homology analysis to ascertain the molecular mimicry between all the TAAs available at cancer peptide database (https://caped.icp.ucl.ac.be/Peptide/list) and viral sequences. The analysis was focused on 99 TAAs binding to HLA-A*0101, 0201, 0301 and 2402 alleles, which altogether cover about 50% of the world population. Several viral sequences (n = 82) sharing homology with the TAAs were identified, but only 20 of such viral sequences are predicted to be SBs to the corresponding MHC-class I alleles. Surprisingly, 45% of these sequences are derived from Gag and Env HIV-1 proteins, suggesting that HIV-1 may provide several TAA-like epitopes. This could explain the epidemiological notion that people living with HIV or AIDS (PLWHA) have a significantly lower incidence of non-viral associated solid tumors compared to the HIV-negative population [45–47].

**Fig. 2:**
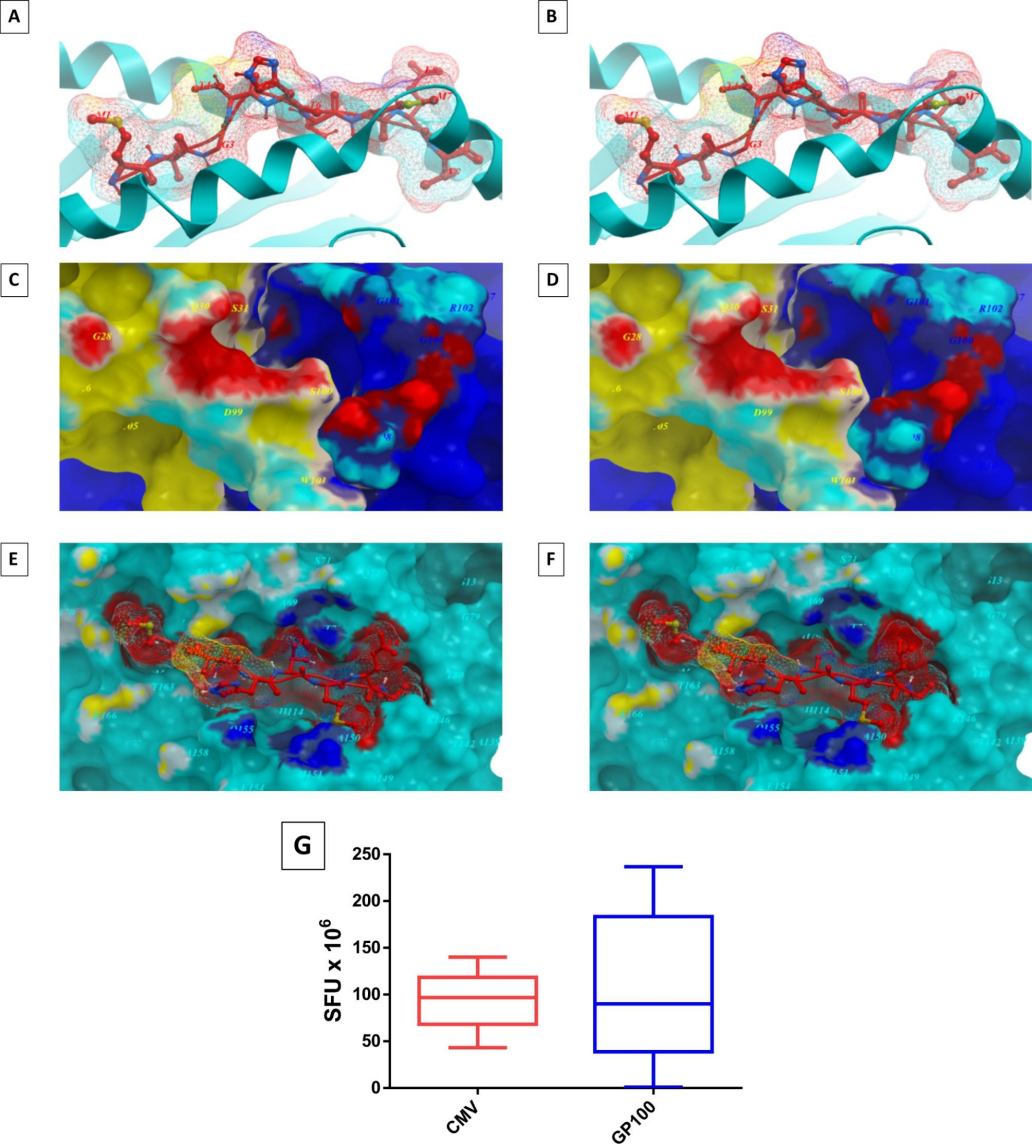
**Molecular mimicry of gp100 and HCMV peptides.** The gp100 MLGTHTMEV peptide and the homologous MLGTHAMLV peptide derived from HCMV are shown bound to the HLA-A*02:01 molecule. The TCR facing residues are presented to the TCR αβ chains with the same conformation (**A**, **B**). The contact areas in red between the two peptides and the TCR αβ chains are identical (**C**, **D**). The areas of contact between the HLA molecule and the TCR αβ chains are identical (yellow and dark blue areas). PBMCs from HLA-A*02:01 positive healthy subjects were immunized ex vivo with HCMV peptide. After 14 days, IFNγ EliSpot assay was performed restimulating the cells with the same viral peptide or with the paired gp100 peptide. SFU = IFNγ spot forming units (**G**)

Moreover, most of the paired TAA and viral epitopes not only share the same conformation but also the same contact patterns with the HLA-A*0201 molecule as well with the TCR α and β chains. The best examples are represented by the gp100/HCMV, the HEPACAM/ENCEPH, the CD274/HIV, Tyrosinase/HIV, CEA/HIV and Telomerase/HIVa pairs for which the contact pattern with the HLA-A*0201 as well as the TCR α and β chains are identical. The biological confirmation of cross-reactive T cell responses against the paired peptides was assessed by ex vivo immunization experiments, showing induction of a significant cross-reactive response within the paired peptides [48] (Fig. 2).

**Fig. 3:**
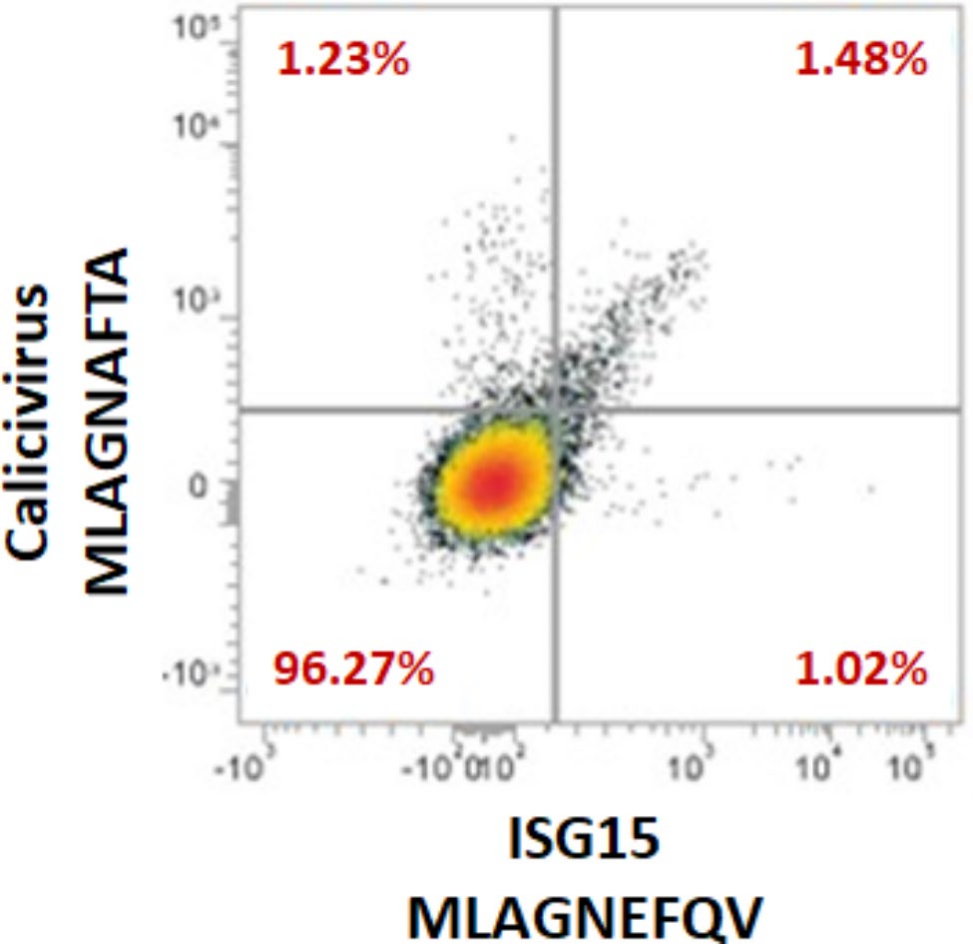
**T cell cross reactivity against homologous peptides.** T cells from HCC patient show cross-reactivity with the HCC-specific MLAGHEFQV peptide and the homologous MLAGNAFTA peptide derived from Human Calicivirus. Results were obtained with MHC-class I tetramers for HLA-A*02:01 loaded with the indicated peptides. (modified from Cavalluzzo et al., 2021)

In addition, we have recently identified novel non-mutated shared specific target antigens from protein highly expressed in hepatocellular carcinoma (HCC) with high sequence and structural homology to viral epitopes. These derive from Influenza virus, hepatitis C virus (HCV), hepatitis B virus (HBV), adenovirus, human cytomegalovirus (HCMV) and human calicivirus. In most cases, the paired peptides show similar contact patterns with the TCR α and β chains and T cell cross-reactivity was confirmed by tetramer binding assay with PBMCs from both HCC patients and healthy individuals. The highest percentage of CD8 + T cells reactive to a viral epitope and, consequently, cross-reactive against the paired epitope was observed for the peptide derived from the human calicivirus, which is one the most frequent causes of acute gastroenteritis [49] (Fig. 3).

Finally, we have identified peptides derived from HIV-1 with high sequence and conformational homology to peptides derived from cancer-specific proteins in colon and breast cancers, strong binders to 12 HLA alleles that altogether cover more than 70% of the World population (http://www.allelefrequencies.net). The cross-reactivity to HIV and TAA peptides was ultimately demonstrated in ex vivo binding assays, showing that only PBMCs from HIV-positive patients, and not those from HIV-negative controls, reacted against HLA-matched TAAs [50] (Table 1).

**Table 1:** Examples of homologies between tumor-associate antigens (TAAs) and viral antigens

HOMOLOGY BETWEEN TAAs AND VIRAL ANTIGENS							
CANCER TYPE	**TAA**		**MoA**		**HLA**	**Evidence**	**Reference**
Melanoma	MART-1_27−35_	AAGIGILTV	HSV-1	GIGIGVLAA	A*02:01	Cross-reactive CTL in melanoma patients	[39]
	MART-1_26−35_	EAAGIGILTV	Pseudorabies virus	VIAGIGILAI	A*02:01	Cross-reactive CTL in melanoma patients	[41, 42]
			Variola virus	MIAGIGISLI	A*02:01		
Lung ca.	TMEM161A	ALGGLLTPL	EBV	CLGGLLTMV	A*02:01	Cross-reactive CTL in lung cancer patients	[43]
Melanoma	Gp100	MLGTHTMEV	HCMV	MLGTHAMLV	A*02:01	Cross-reactive T cells in ex vivo experiments	[48]
Multiple ca.	CD274	LLNAFTVTV	HIV-1	LLNAFAIAV	A*02:01		
Liver ca.	HEPACAM	RLAPFVYLL	Encephal. virus	RLAPFGYKI	A*02:01		
Gut ca.	CEA	IMIGVLVGV	HIV-1	IMVGALIGV	A*02:01		
Multiple ca.	TELOMERASE	RLVDDFLLV	HIV-1	RLVDEFLAI	A*02:01		
Liver ca.	ISG15	MLAGNEFQV	Calicivirus	MLAGNAFTA	A*02:01	Cross-reactive T cells in ex vivo experiments	[49]
	MDK	ALLALTSAV	HCV	ALMAFTSAV	A*02:01		
	MDK	LLLTLLALL	Adenovirus	LLLTLLLLL	A*02:01		
	C1QTNF12	LLGPQLVLL	HBV	LLGPLLVLL	A*02:01		
Breast ca.	CCR9	AIADLLFLV	HIV-1	RLRDLLFLV	A*02:01	Cross-reactive T cells in ex vivo experiments	[50]
Colon ca.	EPCAM	VVAGIVVLV		VVAGIIALV	A*02:01		
Brain ca.	DPYSL2	IPRRTTQRI		IPRRIRQRI	B*07:02		
Lung ca.	CNIH4	LLNLPVATW		HCNLSVATW	B*58:01		
Prostate ca.	NDUFS2	VSDGSSRPY		SSDNSSRPY	A*01:01		

When multiple MoAs have been identified with homology to the same TAA, the most representative has been reported in the table. The full list is available at the indicated reference.

## TAAs and bacterial antigens

Likewise to homology with viral peptides, peptides with homology to MART-1_27−35_ have been originally identified in different bacteria strains [39, 41]. MART-1-specific T cells have been shown to cross-react and kill cells loaded with homologous bacterial peptides which, although different in the two studies, share the highly conserved motif P_2_ – AGIGI - P_7_ with the MART-1_(27−35)_ epitope. A MHC class II-associated peptide from the permease protein of the *Mycoplasma penetrans* HF-2 bacterium (HF-2_216–229_) has been identified with a high-degree of structural homology with the TAA MAGE-A6_172–187_. CD4^+^ T cells cross-reacting to both peptides have been identified in healthy donors and melanoma patients [51]. The same sequences have been subsequently shown to include a MHC class I-associated peptide recognized by cross-reactive CD8^+^ T cells in healthy individuals and melanoma patients. Response to the HF-2_216–229_ peptide was qualitatively and quantitatively comparable in healthy donors and in melanoma patients. On the contrary, response to MAGE-A6_172–187_ was significantly stronger in healthy donors, suggesting that the limited T cell response in melanoma patients could explain the cancer progression [52].

**Fig. 4:**
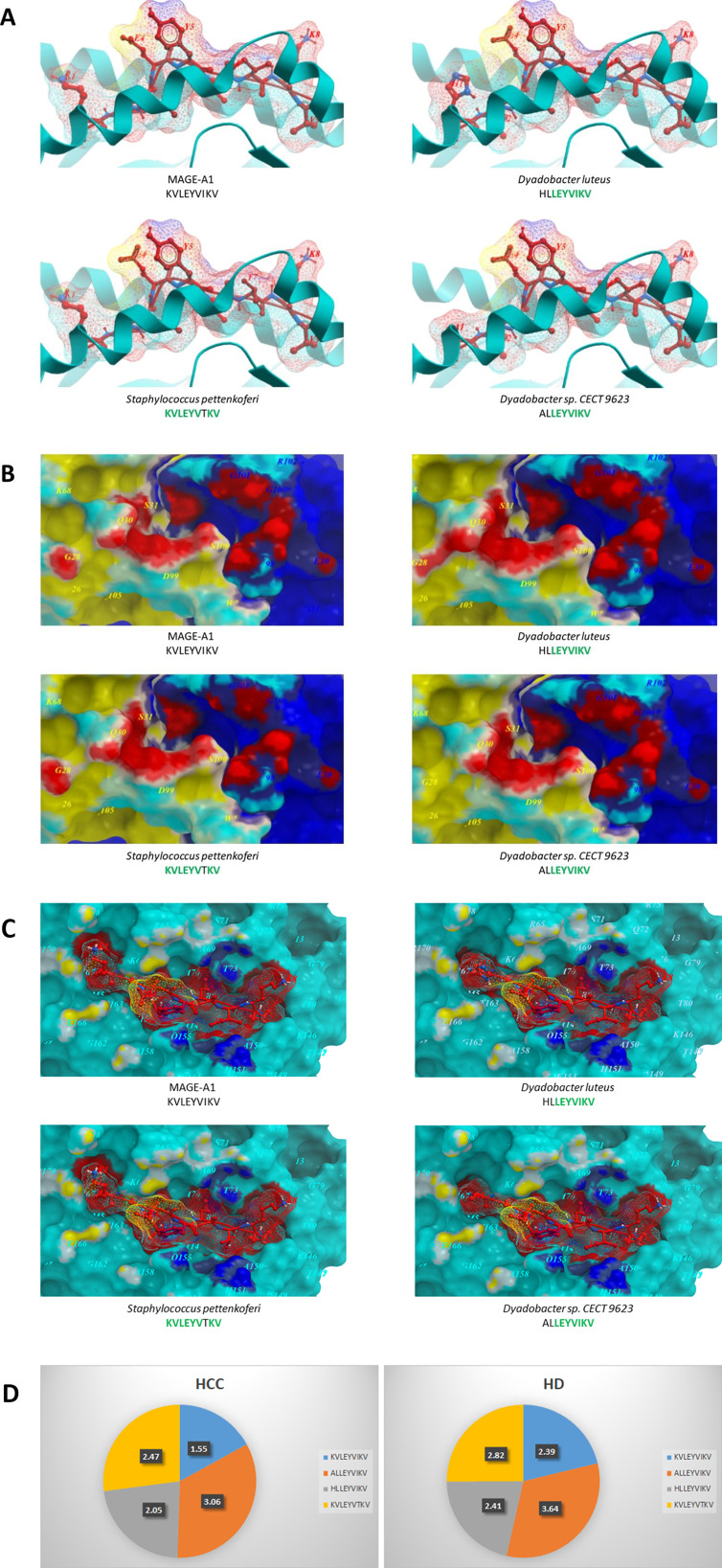
**Molecular mimicry of MAGE-A1 and microbiota peptides.** The MAGE-A1_278 − 286_ KVLEYVIKV peptide and the homologous peptides derived from the indicated microbiota bacteria are shown bound to the HLA-A*02:01 molecule. (**A**) The TCR facing residues are presented to the TCR αβ chains with the same conformation. (**B**) The contact areas in red between the peptides and the TCR αβ chains are mostly identical. (**C**) The areas of contact between the HLA molecule and the TCR αβ chains are identical (yellow and dark blue areas). (**D**) CD8^+^ T cells from a HCC patient (HCC) and a healthy donor (HD) were incubated with the MAGE-A1_278 − 286_ KVLEYVIKV peptide and the indicated homologous peptides derived from microbiota bacteria. Values represent the level of CD8^+^ T cell activation expressed as absolute value of fold-increase

The tail length tape measure protein (TMP) of a prophage found in the genome of the bacteriophage *Enterococcus hirae* includes the H-2K^b^–restricted TSLARFANI peptide, which share a strong homology to the peptide GSLARFRNI derived from the proteasome subunit beta type-4 (PSMB4) protein. This is overexpressed in MCA205 sarcoma cells, whose growth in mice was inhibited by T cells elicited by the TMP peptide. Likewise, the same TMP includes the HLA-A*02:01restricted KLAKFASVV peptide, which share a strong homology to the peptide KLQKFASTV derived from the glycerol-3-phosphate dehydrogenase 1-like (GPD1-L) protein. CD8 + T cells cross-reactive to both peptides were found in three out of six non-small-cell lung cancer (NSCLC) patients [53], The commensal bacterium *Bifidobacterium breve (B. breve)* includes the H-2K^b^–restricted SVYRYYGL peptide, which is able to elicit T cells cross-reacting with the neoepitope antigen SIYRYYGL expressed in engineered B16.SIY cell line. Tumor growth in mice was inhibited by gut colonization with *B. breve* or after adoptive transfer of K^b^SVY-expanded T cells [54]. In this regards, we have very recently shown that homology between TAAs and peptides from microbiota species of the *Firmicutes* and *Bacteriodetes* phyla is a frequent finding [55]. This has a potential enormous relevance in cancer immunology, given that the two phyla together account for > 90% of the human gut microbiota [56]. The vast majority (about 70%) of the TAA-microbiota paired epitopes share 6–7 identical residues along the sequence and three of them are identical. The consensus of the microbiota-derived epitopes matches with the sequence of the corresponding TAA and the amino acid substitutions at each position are mostly conservative, with limited impact on the charge of the peptide.The structural conformation of the paired peptides is highly similar or even identical and the spatial conformation of TCR-facing residues can be identical with the same values of planar as well as dihedral angles. Moreover, predicted contact areas with both HLA and TCR chains are mostly indistinguishable, suggesting that the paired peptides may be recognized by cross-reacting T cells (Fig. 4).

Preliminary results show that CD8^+^ T cells recognize microbiota-derived peptides, confirming that indeed they are naturally presented to the immune system in association with MHC class I molecules. Significant level of cross-reactivity to highly homologous TAAs and microbiota-derived peptides was observed in both healthy donors and cancer patients, suggesting that anti-microbial CD8 + T cells may bind to TAAs expressed by cancer cells and play a key role in controlling tumor growth. Interestingly, CD8^+^ T cell reactivity against TAAs was lower in cancer patients in support of poor efficacy of the anti-tumor immune response (Fig. 4D) (Table 2).

**Table 2:** Examples of homologies between tumor-associate antigens (TAAs) and bacterial antigens

HOMOLOGY BETWEEN TAAs AND BACTERIAL ANTIGENS							
CANCER TYPE	**TAA**		**MoA**		**HLA**	**Evidence**	**Reference**
Melanoma	MART-1_27−35_	AAGIGILTV	Bacillus polymyxa	GAGIGVLTA	A*02:01	Cross-reactive CTL in melanoma patients	[39]
			E. coli	QAGIGILLA	A*02:01		
	MART-1_26−35_	EAAGIGILTV	Chl. trachomatis	MLSGIGIFFI	A*02:01	Cross-reactive CTL in melanoma patients	[41]
	MAGE-A6_172 − 187_	YIFATCLGLS	Myc. penetrans	YIFAACLLLI	A*02:01	Cross-reactive CTL in melanoma patients	[51]
Multiple ca.	MAGE-A10	GLYDGMEHL	Cohnella nanjingensis	GLYDGVEHV	A*02:01	Bioinformatics analyses	[53]
			Chitinophagaceae bacterium	GMYDGLEHV	A*02:01		
	MAGE-A3	KVAELVHFL	Sedimentibacter sp.	KIAELVHFL	A*02:01		
			Solirubrum puertoriconensis	KVAELVHHV	A*02:01		
	MAGE-A1	KVLEYVIKV	Firmicutes bacterium	KVLEYVIRV	A*02:01		
			Candidatus Faecivivens stercorigallinarum	KVLEYLPKV	A*02:01		
	MAGE-C2	ALKDVEERV	Loigolactobacillus jiayinensis	ALSDVEERV	A*02:01		
			Lachnospiraceae bacterium MD329	ALNDVEERL	A*02:01		
	MAGE-A12	FLWGPRALV	Bacteroidales bacterium	FLWGSIALV	A*02:01		
			Clostridia bacterium	FLWGPFSLV	A*02:01		
	SSX-2	KASEKIFYV	Lachnospiraceae bacterium	KAAEKFFYV	A*02:01		
			Agathobacter sp.	KASDKIFYV	A*02:01		
	MAGE-C1	KVVEFLAML	Clostridia bacterium	KILEFLAML	A*02:01		
			Clostridia bacterium	KVVEFLAML	A*02:01		

When multiple MoAs have been identified with homology to the same TAA, the most representative has been reported in the table. The full list is available at the indicated reference.

## Conclusions

Increasing but not definitive evidences support the concept that the molecular mimicry, originally described playing a role in the pathogenesis of autoimmune diseases, is relevant in shaping the anti-tumor T cell repertoire.

The sequence homology between tumor associated antigens and antigens derived from microorganisms (viruses and bacteria) can be very high, resulting in stretches of 6–9 identical aligned amino acids (66.6 to 100% homology). Given that the probability of such event is extremely low (1.56 × 10^− 8^ − 1.95 × 10^− 12^), the frequency of such a finding for several paired TAAs and microorganisms strongly suggests a biological relevance in ruling the tumor-host interaction. Ultimately, this may possibly explain the existence itself of the human race.

Indeed, although cancer cells may frequently arise in human bodies exposed to environmental carcinogens, only in few cases such event progresses into tumor formation. One of the possible explanation is that tumor growth at primary site is inhibited at the very early stages (T_is_ or T_0_) in the vast majority of cases, when the immune suppressive TME is not yet fully established and the immune system can efficiently exert its anti-tumor effect. But, this can be achieved only if a memory CD8 + T cell immunity specific for TAAs is already established and prompt to expand upon development of cancer cells. Nowadays, it is conventionally accepted that such a condition can only occur if induced by a preventative cancer vaccine, which is currently available only for virus-induced cancers (e.g. HBV and HPV) [57].

However, given the homology between TAAs and antigens derived from microorganisms, individuals may be considered undergoing “natural” preventative cancer vaccines every time they are exposed to pathogens (i.e. viruses and bacteria) as well as intestinal microbiota.

The wide pool of memory CD8^+^ T cell clones specific for the MoAs may promptly expand if elicited by a homologous TAA expressed by cancer cells in a nascent tumor lesion. The same mechanism may result in a pool of anti-TAA memory CD4^+^ T cells elicited by homologous longer (15 mers) MoAs and ready to expand for regulating the quality, magnitude, and durability of the effector CD8^+^ CTL-mediated immunity in vivo [51, 52]. This would stop the cancer immunoediting process at the Elimination stage, preventing the tumor from entering the Equilibrium and the Escape stages (the three Es of the cancer immunoediting) [58].

The possible role of the molecular mimicry and cross-reactive T-cells in the spontaneous regression of tumors has been proposed but never experimentally demonstrated [59, 60]. Moreover, several studies have reported that gut microbiome influences the response to immune checkpoint inhibitors (ICIs) but also the survival in pancreatic cancer after surgery [61–66]. However, none of these studies has supported these observations with experimental data confirming molecular mimicry of microbiota-derived antigens and TAA as well as cross-reactive T cells.

Collectively, the most recent data reported by our groups and others show convincing data on high sequence and structural homology between TAAs and MoAs as well as cross-reactive CD8^+^ T cells with potential strong anti-tumor efficacy.

In summary, a growing body of evidence suggests that the broadness of the anti-MoA memory CD8 + T cell repertoire - built upon the individual experience of interaction with intracellular pathogens and intestinal microbiota - may strongly influence the fate of tumor progression and prognosis. Indeed, a broader number of cross-reactive TCRs elicited by MoAs during one’s lifetime is correlated with a greater probability of eliminating cancer cells at very early stages, thereby preventing tumor progression.

More importantly, this might represent an unexplored opportunity to develop next generation anti-cancer therapeutic vaccines. Indeed, the identification of MoAs with high degree of homology with TAAs opens a new frontier in the quest for the optimal public antigens for developing effective preventive as well as therapeutic cancer immunotherapies. Indeed, MoAs do not suffer from immunological tolerance and are highly immunogenic.

In particular, MoAs with high homology to TAAs highly expressed in several cancer types may be employed in multi-targeting preventive as well as therapeutic cancer vaccines. MoAs could be useful in preventive/therapeutic strategies developed for any cancer setting with fully characterized homologous TAAs [67]. The ideal situation would be the inclusion of preventive strategies based on MoAs in pediatric/adolescent vaccine programs, as for preventive HBV and HPV vaccines. Tumor would be inhibited at the very early stages (T_is_ or T_0_). As initial proof-of-concept of protection from cancer, they could be implemented as preventive strategy in populations at high-risk for cancer development (e.g., patients with cirrhotic liver or with cervical intraepithelial neoplasia (CIN)). Needless to say that, if adopted as therapeutic strategy, cancer vaccines based on MoAs would always be combined with systemic therapies aiming at reshaping and overcoming the immunosuppressive tumor microenvironment.

**Fig. 5:**
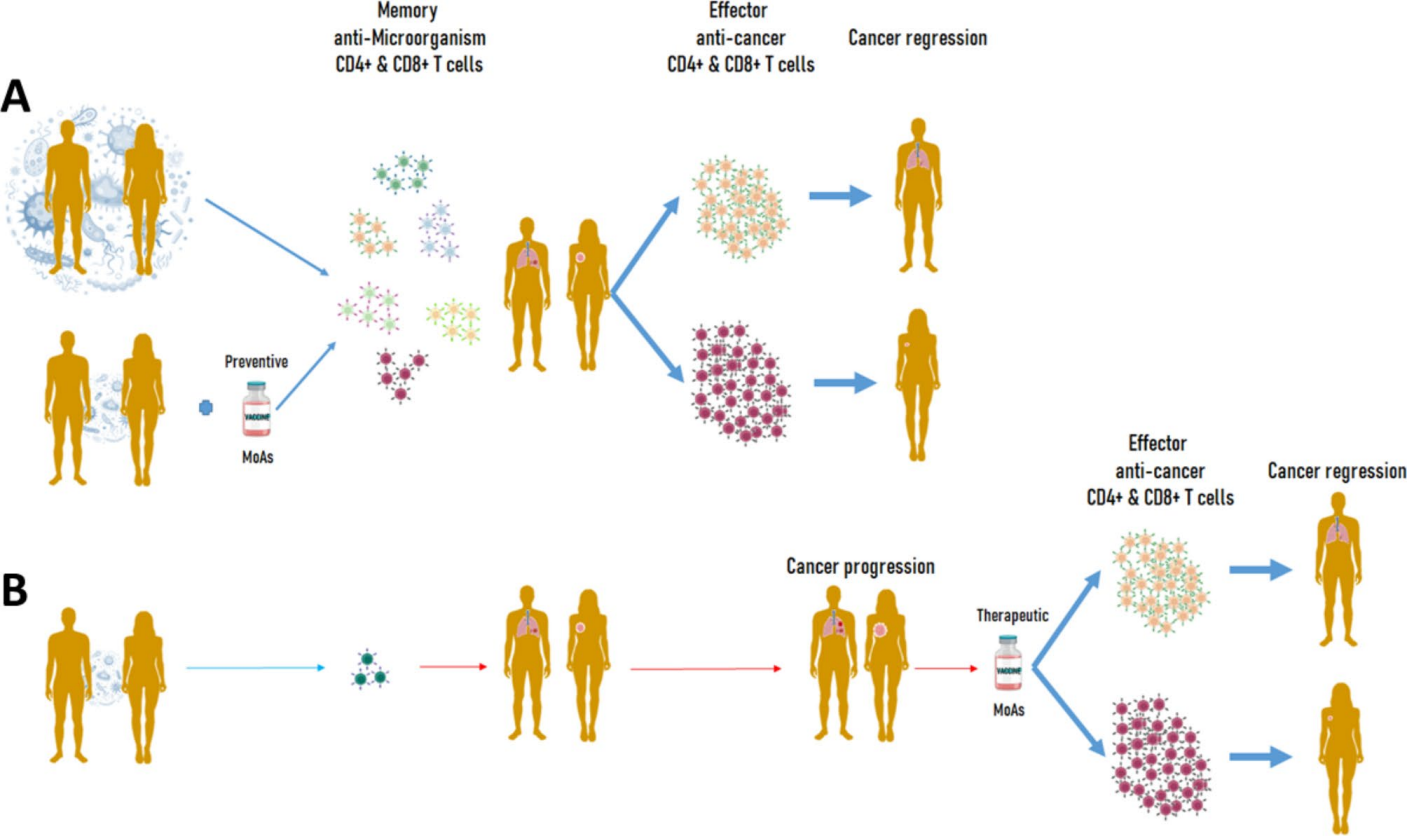
**Microorganism-derived antigens (MoAs) as anti-cancer strategy.** (**A**) The exposure to a large number of microorganisms or the combination with preventive vaccines based on MoAs eventually lead to a broad memory T cell repertoire. This is promptly activated by a tumor presenting shared antigens leading to cancer regression. (**B**) The exposure to a limited number of microorganisms elicits a narrow memory T cell repertoire and a cancer may develop and progress. A therapeutic vaccine based on MoAs may elicit an effector anti-cancer T cell response against shared tumor antigens leading to cancer regression

Ultimately, this may represent a turning point in cancer immunotherapy and offer more effective therapies for cancer patients (Fig. 5).

## Data Availability

Data and material will be deposited and publicly available.

## Abbreviations

TAAs: Tumor associated antigens
HLA: Human leukocyte antigen
MoAs: Microorganism antigens
TSAs: Tumor specific antigens
TCR: T cell receptor
MHC-I: Major histocompatibility complex class-I
MAGE: Melanoma associated gene
MART-1: Melanoma-associated antigen recognized by T-cells
HSV-1: Herpes simplex virus-1
HSV-2: Herpes simplex virus-2
PBMCs: Peripheral blood mononuclear cells
CTL: Cytotoxic lymphocyte
TMEM161A: Trans-membrane protein 161 A
EBV: Epstein-Barr Virus
HIV-1: Human immunodeficiency virus type-1
AIDS: Acquired immunodeficiency syndrome
PLWHA: People living with HIV/AIDS
HCMV: Human cytomegalovirus
ENCEPH: Encephalomyelitis virus
CEA: Carcinoembryonic antigen
HCV: Hepatitis C virus
HBV: Hepatitis B virus
HCC: Hepatocellular carcinoma
TME: Tumor microenvironment
ICI: Immune checkpoint inhibitors
TMP: Tail length tape measure protein
GPD1-L: Glycerol-3-phosphate dehydrogenase 1-like
NSCLC: Non-small-cell lung cancer
CIN: Cervical intraepithelial neoplasia
